# Supplementary material for: A latitudinal gradient of deep-sea invasions for marine fishes
Source: Nat Commun. 2023 Feb 11;14:773. doi: 10.1038/s41467-023-36501-4 (PMC9922314; doi:10.1038/s41467-023-36501-4)
Supplement: Supplementary file 1 — Supplementary Information [file 41467_2023_36501_MOESM1_ESM.pdf]

A latitudinal gradient of deep-sea invasions for marine fishes

Supplemental Materials

Table S1 Best-fit all-rates-different Q matrix detailing the transition rates between each discrete state

	polar deep	polar intermediate	polar shallow	temperate deep	temperate intermediate	temperate shallow	tropical deep	tropical intermediate	tropical shallow
polar deep	-0.075	0.016	0.000	0.015	0.016	0.000	0.025	0.004	0.000
polar intermediate	0.015	-0.080	0.007	0.016	0.042	0.000	0.000	0.000	0.000
polar shallow	0.000	0.014	-0.043	0.009	0.009	0.005	0.000	0.007	0.000
temperate deep	0.005	0.000	0.000	-0.050	0.017	0.000	0.019	0.009	0.000
temperate intermediate	0.000	0.007	0.000	0.005	-0.047	0.027	0.000	0.007	0.000
temperate shallow	0.000	0.000	0.000	0.000	0.010	-0.019	0.000	0.000	0.009
tropical deep	0.000	0.000	0.000	0.010	0.000	0.000	-0.017	0.007	0.000
tropical intermediate	0.000	0.000	0.000	0.001	0.005	0.001	0.003	-0.024	0.014
tropical shallow	0.000	0.000	0.000	0.000	0.000	0.002	0.000	0.003	-0.005

Table S2 Summary table for each family in the dataset. Includes the number of species in the dataset for each family (n_species), percent species sampled compared to all known species in that family, median latitude, and the percentage of sampled species in each depth category.

Family	n_species	% cover	median lat	shallow	intermediate	deep
Acanthuridae	59	70.2	-1.8	96.6	3.4	0.0
Agonidae	32	59.3	53.2	25.0	71.9	3.1
Antennariidae	26	54.2	-2.0	80.8	19.2	0.0
Apogonidae	64	17.9	-1.5	98.4	1.6	0.0
Balistidae	30	69.8	-1.0	90.0	10.0	0.0
Blenniidae	62	15.4	2.9	95.2	3.2	1.6
Bothidae	22	13.3	1.9	36.4	59.1	4.5
Carangidae	87	59.2	1.8	63.2	35.6	1.1
Chaetodontidae	91	68.9	-1.4	93.4	6.6	0.0
Clupeidae	83	44.1	14.2	84.3	15.7	0.0
Exocoetidae	23	32.4	-0.4	100.0	0.0	0.0
Gobiidae	181	9.3	-0.7	97.2	2.2	0.6
Haemulidae	54	40.3	12.0	98.1	1.9	0.0
Holocentridae	41	45.6	-2.0	87.8	12.2	0.0
Labridae	276	49.4	-1.9	97.8	2.2	0.0
Lethrinidae	28	68.3	-1.3	92.9	7.1	0.0
Liparidae	50	11.8	50.8	16.0	34.0	50.0
Lutjanidae	70	61.9	-0.6	74.3	25.7	0.0
Macrouridae	82	21.8	17.0	0.0	29.3	70.7
Monacanthidae	36	33.6	-1.8	80.6	16.7	2.8
Moridae	25	23.1	-1.8	8.0	36.0	56.0
Mullidae	31	31.3	-1.9	87.1	12.9	0.0
Muraenidae	52	25.4	-0.7	82.7	17.3	0.0
Myctophidae	94	37.9	-2.3	0.0	40.4	59.6
Nemipteridae	22	31.0	0.0	86.4	13.6	0.0
Notothenioid	71	NA	-67.0	9.9	71.8	18.3
Ophichthidae	27	8.2	-0.9	77.8	22.2	0.0
Ophidiidae	28	10.8	2.2	25.0	46.4	28.6
Paralichthyidae	24	36.9	21.8	62.5	37.5	0.0
Platycephalidae	23	28.4	-17.7	73.9	26.1	0.0
Pleuronectidae	36	53.7	53.0	8.3	61.1	30.6
Pomacanthidae	50	54.9	0.1	98.0	2.0	0.0
Pomacentridae	217	52.7	-1.9	100.0	0.0	0.0
Psychrolutidae	60	142.9	52.4	28.3	58.3	13.3
Sciaenidae	46	16.1	9.0	89.1	10.9	0.0
Scombridae	32	59.3	3.1	46.9	46.9	6.2
Sebastidae	85	63.9	43.5	21.2	64.7	14.1
Serranidae	181	32.0	6.1	73.5	26.5	0.0
Soleidae	24	13.3	13.9	54.2	41.7	4.2
Sparidae	48	30.4	16.6	66.7	33.3	0.0
Sternoptychidae	21	28.4	-0.7	9.5	42.9	47.6
Stomiidae	36	12.3	1.3	2.8	8.3	88.9
Syngnathidae	50	16.3	-1.7	96.0	4.0	0.0
Tetraodontidae	42	20.8	-0.3	90.5	9.5	0.0
Triglidae	23	18.3	20.9	52.2	47.8	0.0
Zoarcidae	71	24.4	48.2	8.5	46.5	45.1

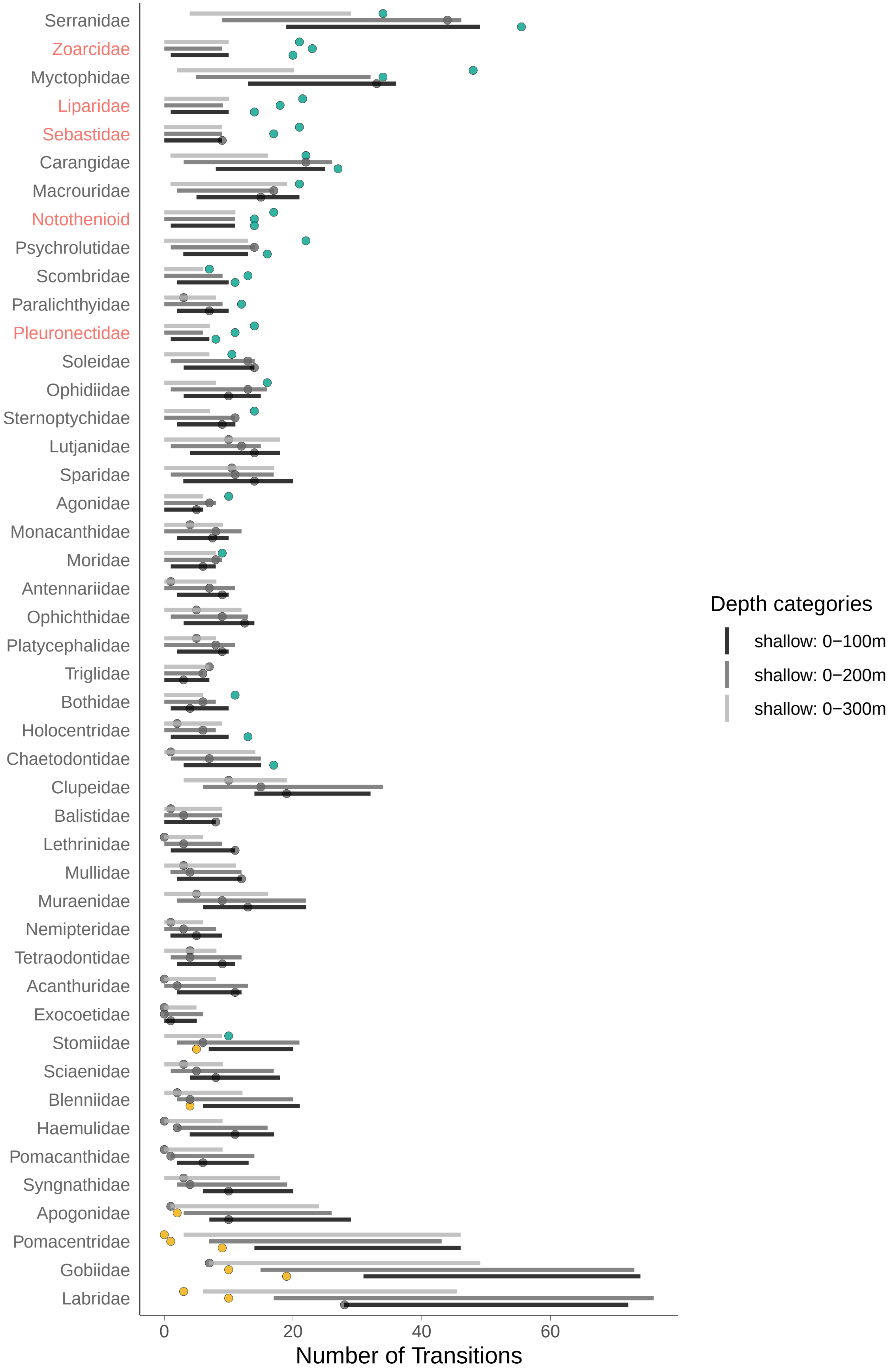
Serranidae
Zoarcidae
Myctophidae
Liparidae
Sebastidae
Carangidae
Macrouridae
Notothenioid
Psychrolutidae
Scombridae
Paralichthyidae
Pleuronectidae
Soleidae
Ophidiidae
Sternoptychidae
Lutjanidae
Sparidae
Agonidae
Monacanthidae
Moridae
Antennariidae
Ophichthidae
Platycephalidae
Triglidae
Bothidae
Holocentridae
Chaetodontidae
Clupeidae
Balistidae
Lethrinidae
Mullidae
Muraenidae
Nemipteridae
Tetraodontidae
Acanthuridae
Exocoetidae
Stomiidae
Sciaenidae
Blenniidae
Haemulidae
Pomacanthidae
Syngnathidae
Apogonidae
Pomacentridae
Gobiidae
Labridae
0
20
40
60
Number of Transitions
Depth categories
shallow: 0−100m
shallow: 0−200m
shallow: 0−300m

Figure S1 Results of re-running analyses with different depth categories (grey bars). Points represent the observed number of depth transitions averaged across 100 stochastic character maps (green: greater than expectation, grey: within expectation, yellow: below expectation). Findings are consistent across all depth categorization systems.

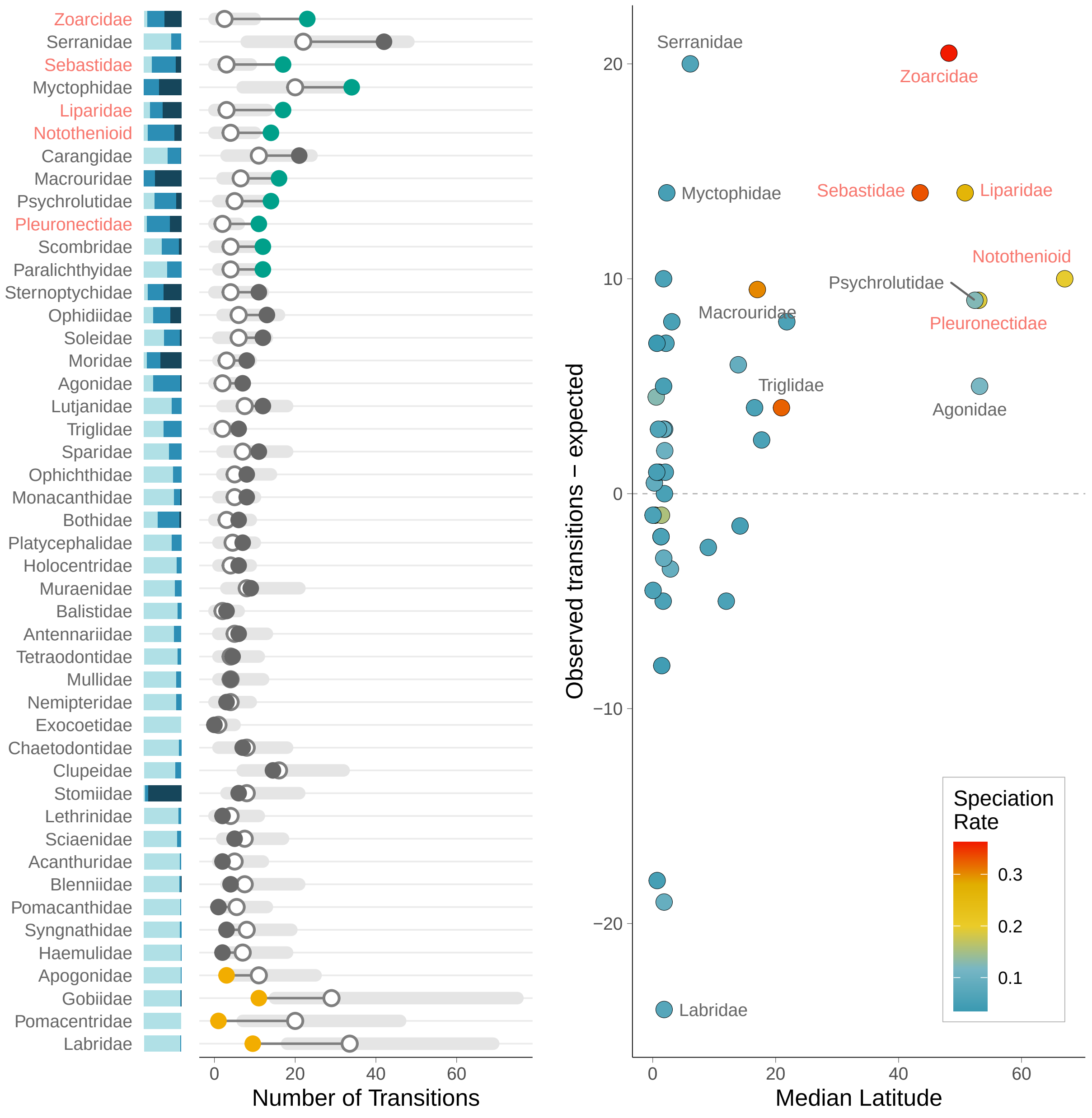

Figure S2 Results of re-running analyses with the Rabosky phylogeny rescaled to the published phylogeny of Alfaro et al. 2018.

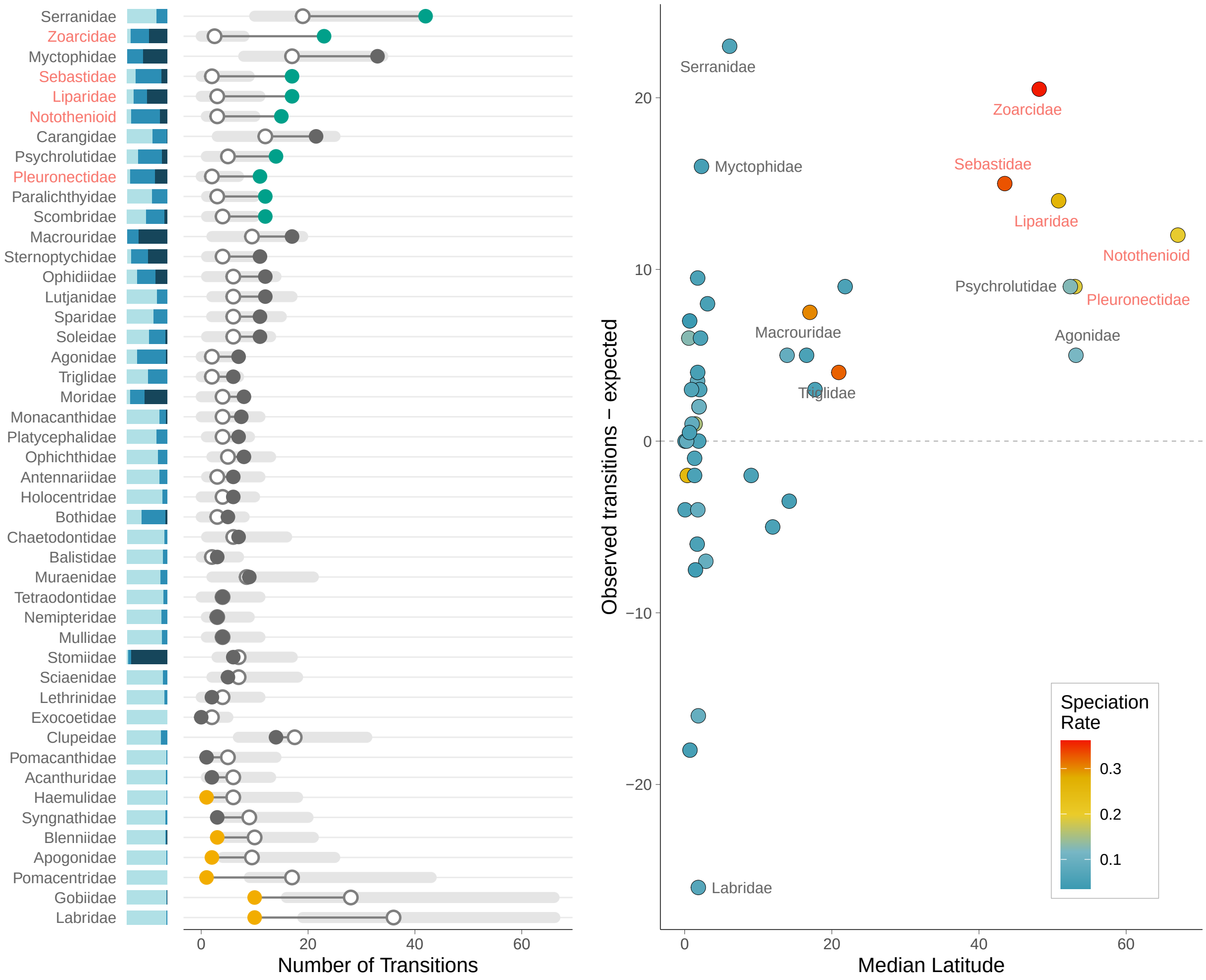

Figure S3 Results of re-running analyses with the Rabosky phylogeny rescaled to the published phylogeny of Ghezelayagh et al. 2022.

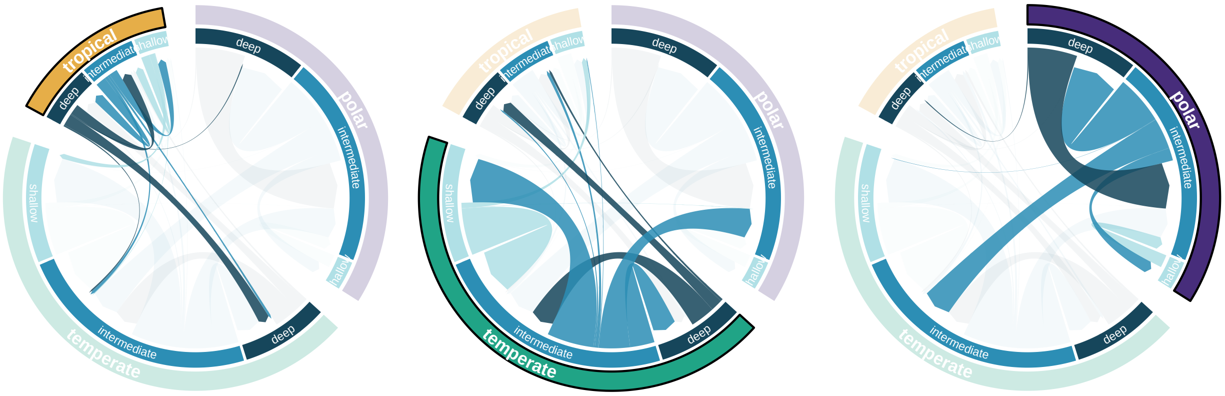

Figure S4 Results of re-running Mk-model analyses with the trimmed UCE phylogeny of Ghezelayagh et al. 2022.

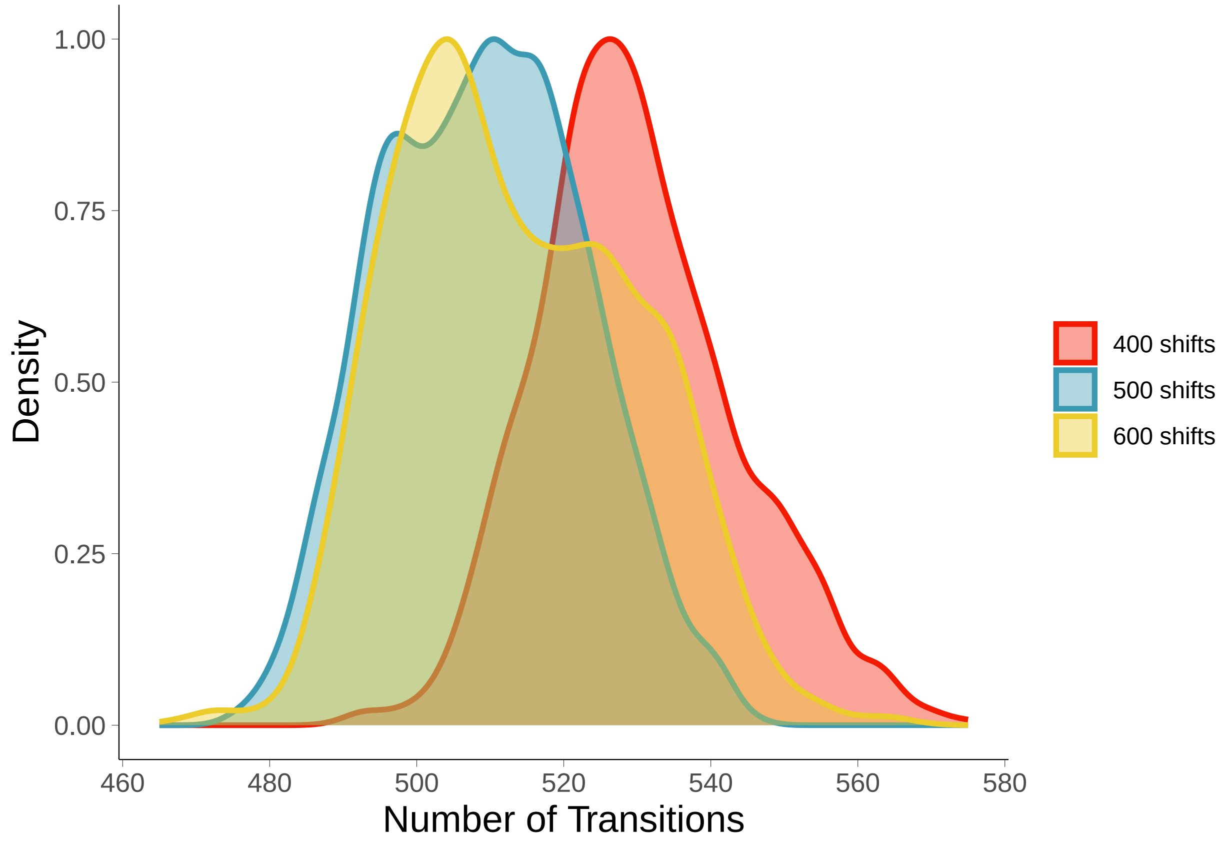

Figure S5 Posterior distribution of the number of discrete state transitions across the phylogeny for MCMCs with different priors.

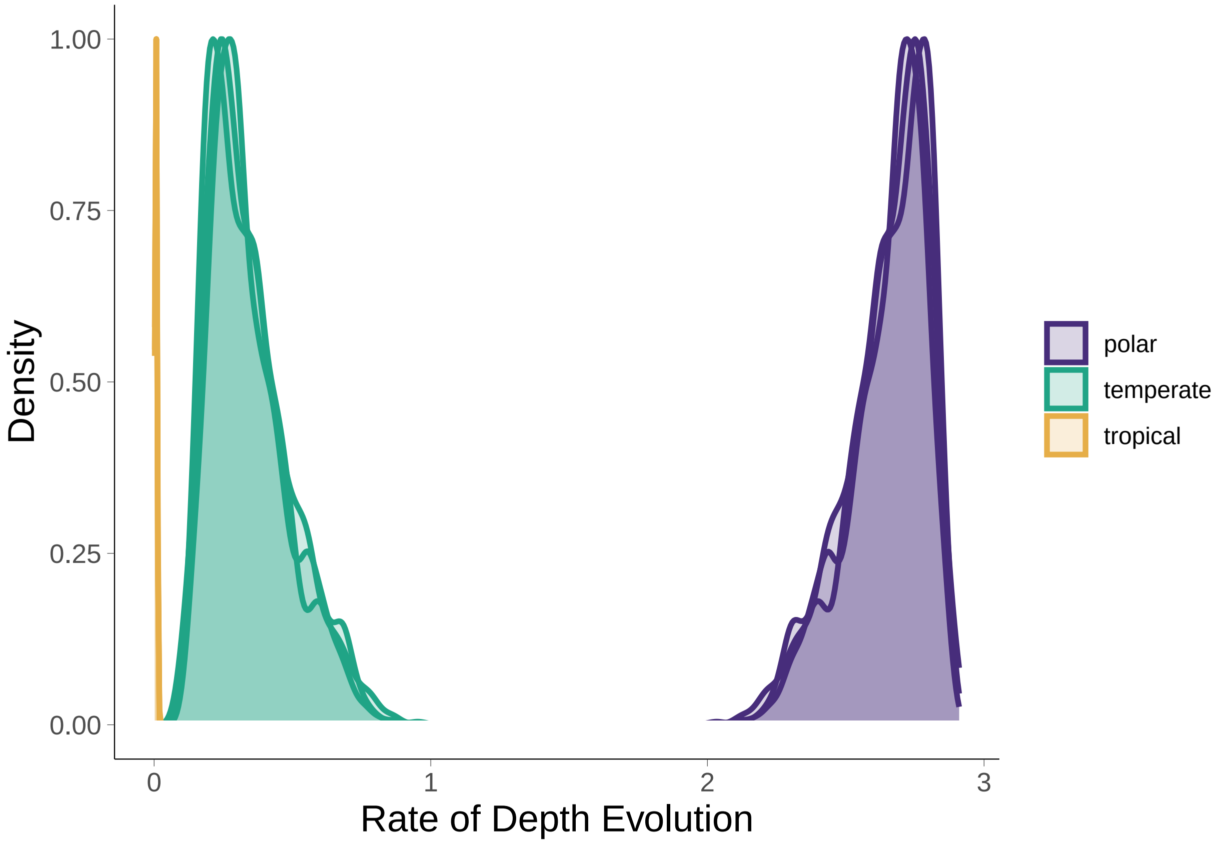

Figure S6 Posterior distributions of state-specific rates of depth evolution for each MCMC chain.

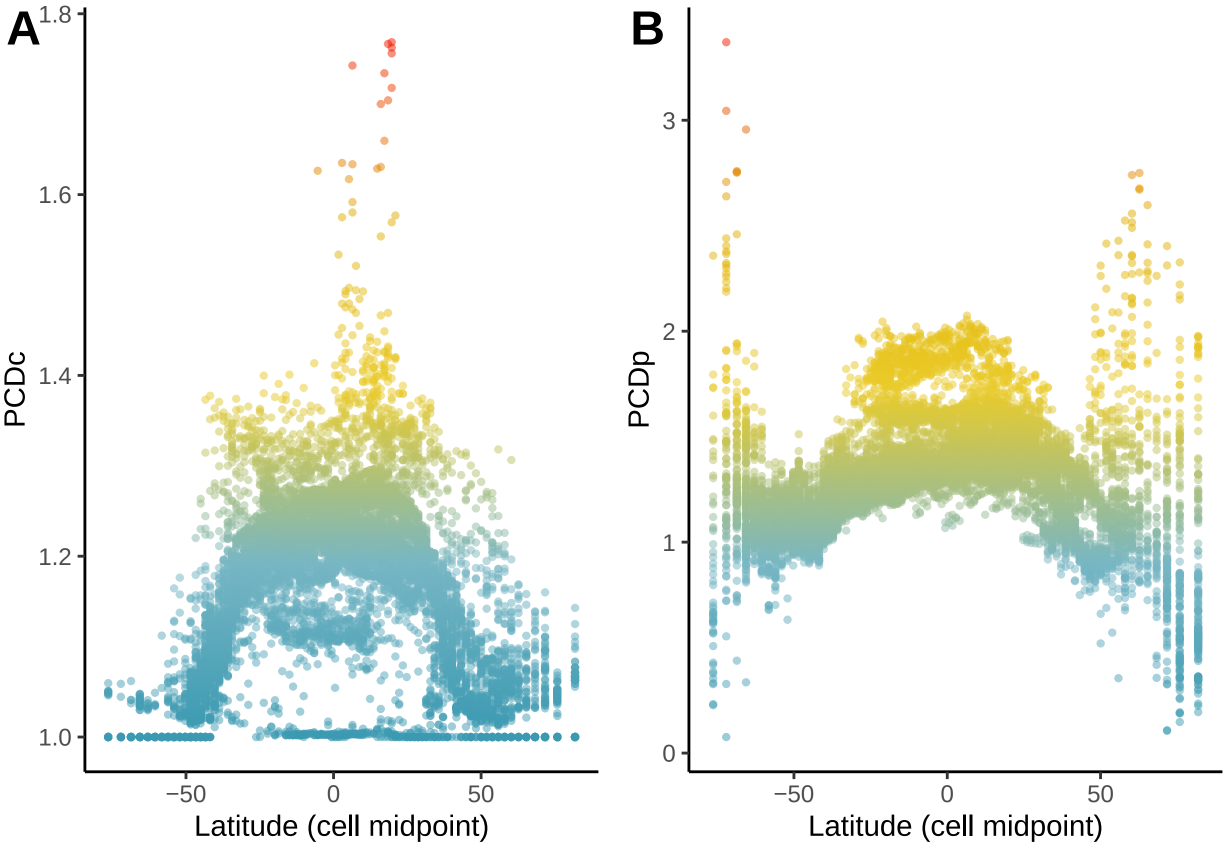

Figure S7 The two components of phylogenetic community dissimilarity, PCDc (Panel A) and PCDp (Panel B), visualized by latitude. PCDc is analogous to Sorenson's Index, while PCDp evaluates the phylogenetic distance between nonshared species in different communities. Shallow and deep fishes at high latitudes are more similar than those at lower latitudes by both metrics.

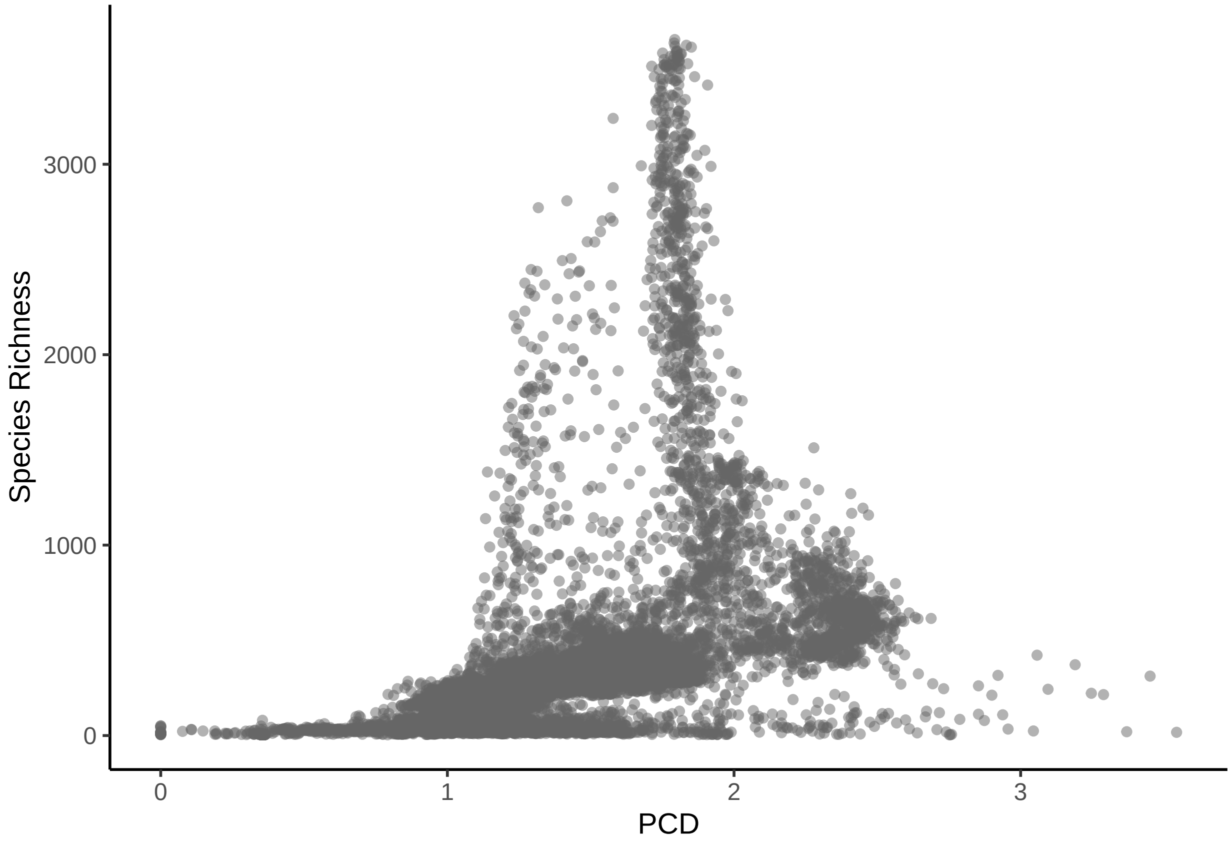

Figure S8 Species richness and phylogenetic community dissimilarity (PCD) within global grid cells are weakly correlated using a two-sided linear regression without adjustments for multiple comparisons ($p < 2e\text{-}16$, r-squared = 0.155).

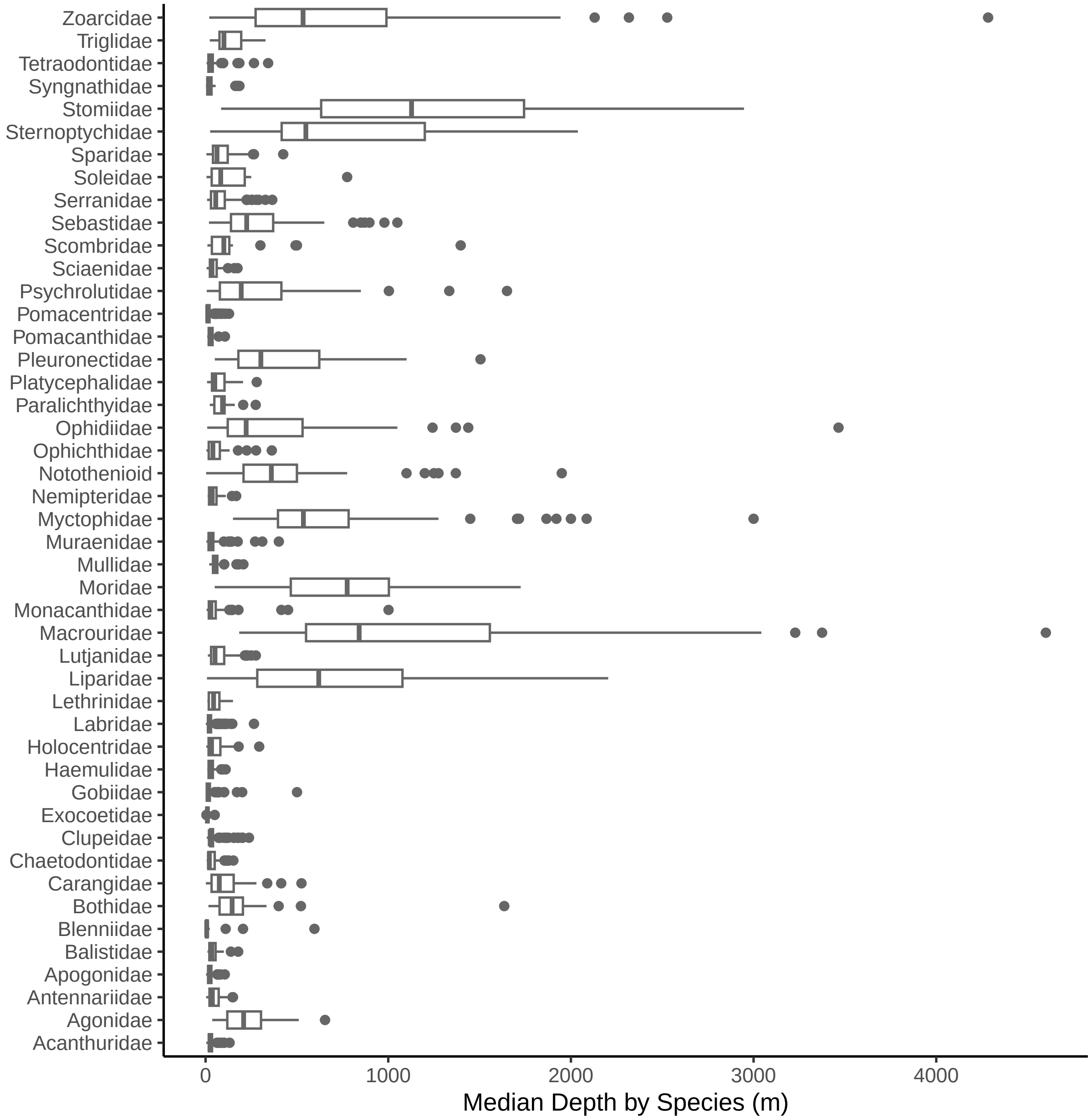

Figure S9 Summary figure of the distribution of depths for species within each of the 46 clades (n = 2,816 species). Each box contains the interquartile range with the median as the vertical bolded line. Whiskers extend to 1.5 times the interquartile range and outliers are plotted as points outside of the range.

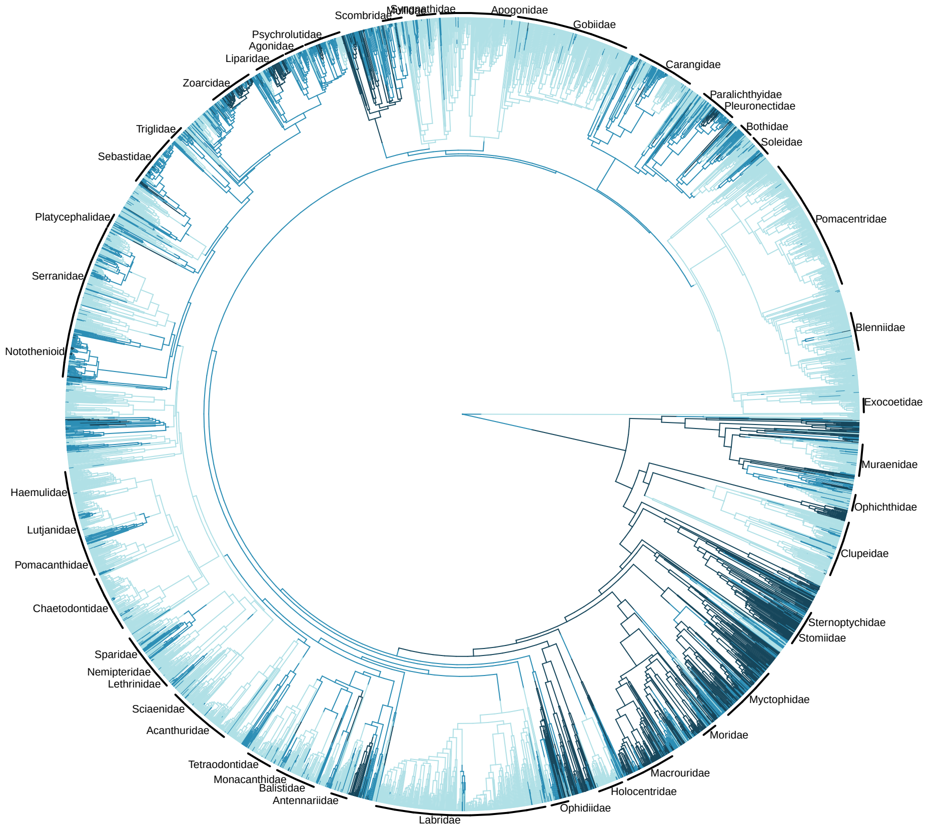

Figure S10 Stochastic character map of depth evolution with all 49 clade names printed at the most recent common ancestor. Darker branch colors represent deeper depths.

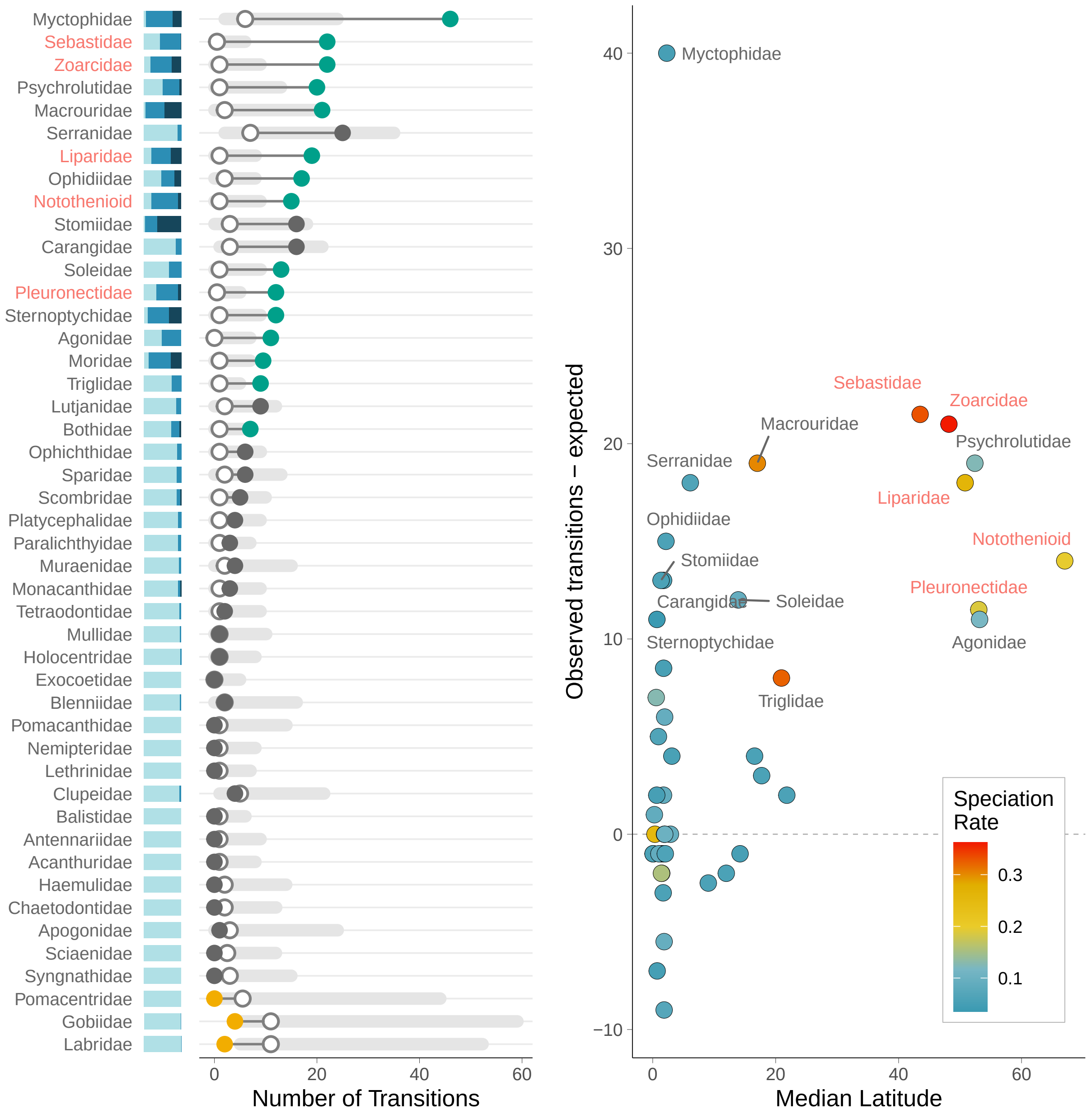

Figure S11 Results of re-running analyses with species categories based on median depth as opposed to maximum depth. Points represent the observed number of depth transitions averaged across 100 stochastic character maps (green: greater than expectation, grey: within expectation, yellow: below expectation). Findings are largely consistent with the original analyses.

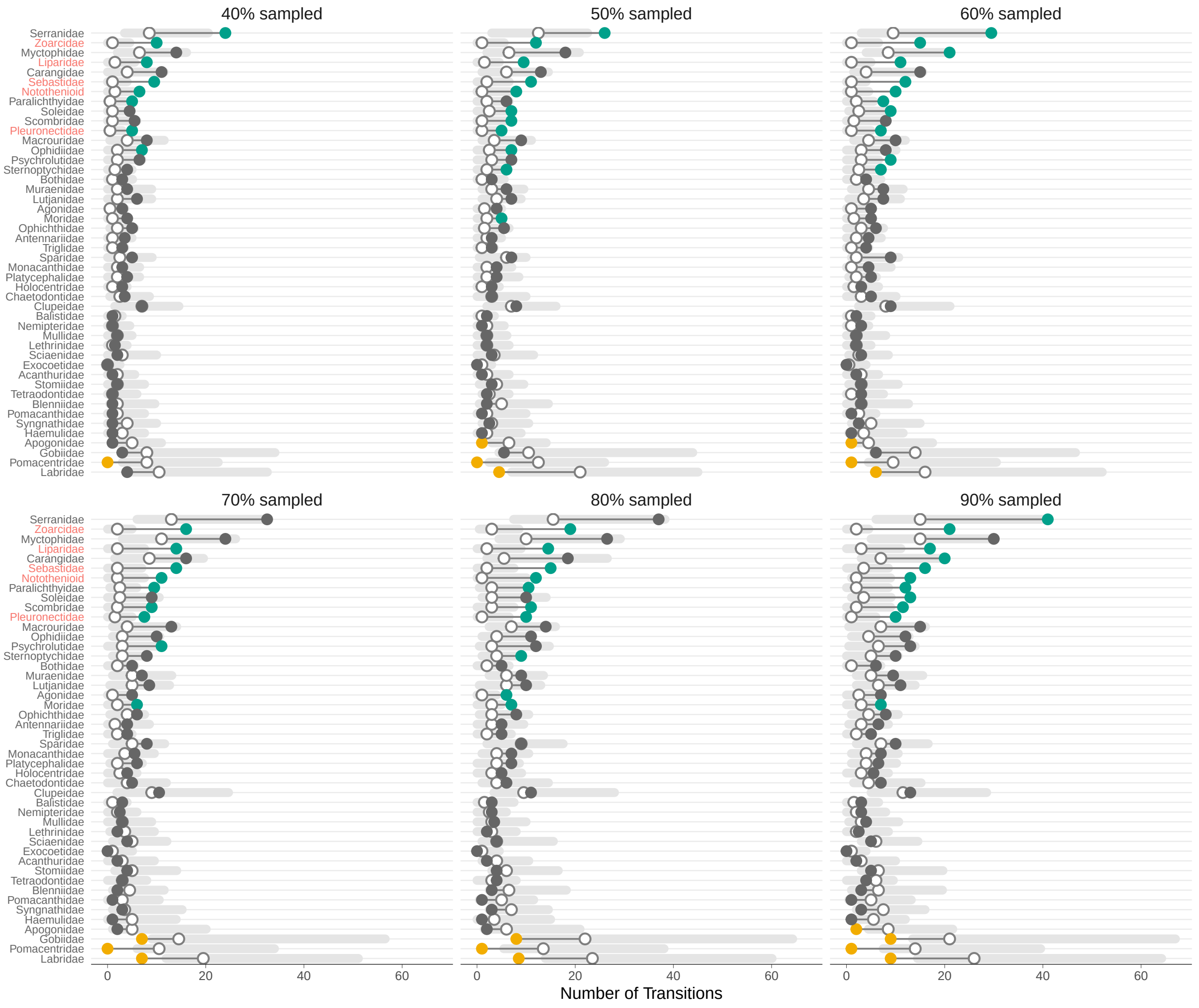

Figure S12 Results of sensitivity analyses with the Rabosky phylogeny randomly re-sampled at 40-90% of tips to approximate topological uncertainty.
