# Supplementary material for: A latitudinal gradient of deep-sea invasions for marine fishes
Source: Nat Commun. 2023 Feb 11;14:773. doi: 10.1038/s41467-023-36501-4 (PMC9922314; doi:10.1038/s41467-023-36501-4)
Supplement: Supplementary file 3 — Description of Additional Supplementary Files [file 41467_2023_36501_MOESM3_ESM.pdf]

## Description of Additional Supplementary Files

File Name: Supplementary Data 1
Description: Table of raw species-level data from Fishbase including recorded depth range and salinity.

File Name: Supplementary Data 2
Description: Random sample of 100 species with Fishbase depth data corroborated by other primary literature.
