# Supplementary material for: A latitudinal gradient of deep-sea invasions for marine fishes
Source: Nat Commun. 2023 Feb 11;14:773. doi: 10.1038/s41467-023-36501-4 (PMC9922314; doi:10.1038/s41467-023-36501-4)
Supplement: Supplementary file 6 — Reporting Summary [file 41467_2023_36501_MOESM6_ESM.pdf]

Corresponding author(s): Sarah Friedman
Last updated by author(s): Jan 11, 2023

Reporting Summary

Nature Portfolio wishes to improve the reproducibility of the work that we publish. This form provides structure for consistency and transparency in reporting. For further information on Nature Portfolio policies, see our Editorial Policies and the Editorial Policy Checklist.

Statistics

For all statistical analyses, confirm that the following items are present in the figure legend, table legend, main text, or Methods section.

n/a	Confirmed	
☐	☒	The exact sample size (*n*) for each experimental group/condition, given as a discrete number and unit of measurement
☒	☐	A statement on whether measurements were taken from distinct samples or whether the same sample was measured repeatedly
☐	☒	The statistical test(s) used AND whether they are one- or two-sided<br>*Only common tests should be described solely by name; describe more complex techniques in the Methods section.*
☒	☐	A description of all covariates tested
☐	☒	A description of any assumptions or corrections, such as tests of normality and adjustment for multiple comparisons
☐	☒	A full description of the statistical parameters including central tendency (e.g. means) or other basic estimates (e.g. regression coefficient) AND variation (e.g. standard deviation) or associated estimates of uncertainty (e.g. confidence intervals)
☐	☒	For null hypothesis testing, the test statistic (e.g. *F*, *t*, *r*) with confidence intervals, effect sizes, degrees of freedom and *P* value noted<br>*Give P values as exact values whenever suitable.*
☐	☒	For Bayesian analysis, information on the choice of priors and Markov chain Monte Carlo settings
☒	☐	For hierarchical and complex designs, identification of the appropriate level for tests and full reporting of outcomes
☒	☐	Estimates of effect sizes (e.g. Cohen's *d*, Pearson's *r*), indicating how they were calculated

Our web collection on statistics for biologists contains articles on many of the points above.

Software and code

Policy information about availability of computer code

Data collection	We did not use software to collect data.
Data analysis	All data were analyzed in R (version 4.0.1) and RevBayes (version 1.1.0). Packages in R used for analyses were phytools(version 1.2-0), geomorph(version 4.0.4), rfishbase(version 4.0.0), and diversitree(version 0.9-16). Scripts for analyses are publicly available at github.com/sfriedman/depth-transitions-paper.

For manuscripts utilizing custom algorithms or software that are central to the research but not yet described in published literature, software must be made available to editors and reviewers. We strongly encourage code deposition in a community repository (e.g. GitHub). See the Nature Portfolio guidelines for submitting code & software for further information.

Data

Policy information about availability of data

All manuscripts must include a data availability statement. This statement should provide the following information, where applicable:

- Accession codes, unique identifiers, or web links for publicly available datasets
- A description of any restrictions on data availability
- For clinical datasets or third party data, please ensure that the statement adheres to our policy

Data on species distributions, rates of evolution, depth and the phylogeny are publicly available in the supplementary materials from https://doi.org/10.1038/s41586-018-0273-1. All other data supporting the findings of this study are available within the paper and its supplementary information files.

Human research participants

Policy information about studies involving human research participants and Sex and Gender in Research.

Reporting on sex and gender	N/A
Population characteristics	N/A
Recruitment	N/A
Ethics oversight	N/A

Note that full information on the approval of the study protocol must also be provided in the manuscript.

Field-specific reporting

Please select the one below that is the best fit for your research. If you are not sure, read the appropriate sections before making your selection.

☐ Life sciences ☐ Behavioural & social sciences ☒ Ecological, evolutionary & environmental sciences

For a reference copy of the document with all sections, see nature.com/documents/nr-reporting-summary-flat.pdf

Ecological, evolutionary & environmental sciences study design

All studies must disclose on these points even when the disclosure is negative.

Study description	We used previously published data of depth, latitude, and speciation rates for 4,067 perciform fishes to test for the relationship between latitude and depth evolution across fishes.
Research sample	Our dataset encompasses information for 4,067 perciform species, which represents the largest sampling of fishes globally with associated phylogenetic information.
Sampling strategy	All perciform species with available phylogenetic information, as well as data on depth and species distribution were included in this study.
Data collection	Data were previously published and no additional data were collected for this study.
Timing and spatial scale	Our data span global extant fish species. All data for this study were collated from previously published sources in Fall of 2020.
Data exclusions	No data were excluded from analyses.
Reproducibility	All evolutionary analyses were repeated with multiple depth schemas, on three different phylogenetic trees and across trees with variable species sampling, all with qualitatively similar results.
Randomization	Randomization was not appropriate for the analyses in this study and thus was not utilized.
Blinding	Blinding was not appropriate for the analyses in this study and thus was not utilized.

Did the study involve field work? ☐ Yes ☒ No

Reporting for specific materials, systems and methods

We require information from authors about some types of materials, experimental systems and methods used in many studies. Here, indicate whether each material, system or method listed is relevant to your study. If you are not sure if a list item applies to your research, read the appropriate section before selecting a response.

Materials & experimental systems

n/a	Involved in the study
☒	☐ Antibodies
☒	☐ Eukaryotic cell lines
☒	☐ Palaeontology and archaeology
☒	☐ Animals and other organisms
☒	☐ Clinical data
☒	☐ Dual use research of concern

Methods

n/a	Involved in the study
☒	☐ ChIP-seq
☒	☐ Flow cytometry
☒	☐ MRI-based neuroimaging
